# In Silico Single-Cell Transcriptomic Analysis Reveals Senescence-Associated Programs’ Progress Along the Normal–Adenoma–Carcinoma Sequence in Colorectal Cancer

**DOI:** 10.3390/ijms27219799

**Published:** 2026-10-31

**Authors:** Xu Zhang, Wangxiong Hu

**Affiliations:** 1 Department of Ultrasound in Medicine, The Second Affiliated Hospital, Zhejiang University School of Medicine, Zhejiang University, Hangzhou 310009, China; 2 Cancer Institute (Key Laboratory of Cancer Prevention and Intervention, National Ministry of Education), The Second Affiliated Hospital, Zhejiang University School of Medicine, Hangzhou 310009, China; 3 Cancer Center of Zhejiang University, Hangzhou 310009, China

**Keywords:** cellular senescence, colorectal cancer, MIF-(CD74+CXCR4), senescence-associated secretory phenotype, single-cell

## Abstract

Colorectal cancer (CRC) develops through a multistep progression from normal colonic mucosa to adenoma and ultimately carcinoma. However, the cell-type-specific senescence-associated states during this transition remain incompletely understood. We performed an integrative analysis of single-cell RNA-sequencing and spatial transcriptomic datasets derived from normal mucosa, adenomatous polyps, and primary CRC tissues. Our analyses revealed a progressive increase in senescence-associated transcriptional scores from normal mucosa through adenoma to carcinoma, with the highest senescence scores observed in malignant tissues. This increase was particularly evident in epithelial cells and monocytes. Cell–cell communication analysis identified enhanced macrophage migration inhibitory factor (MIF) signaling between high-senescence-score cells and B-cell populations, primarily through the MIF–CD74/CXCR4 receptor complex. Spatial transcriptomic analysis further showed that high-senescence-score regions were preferentially localized at or near the tumor invasive margin and in proximity to stromal regions, indicating that senescence-associated programs may contribute to the spatial organization of the CRC microenvironment. Together, these findings support a senescence-centered model of colorectal tumor evolution in which epithelial, myeloid, and lymphoid senescence-associated states progressively accumulate and interact through paracrine signaling networks. The MIF–CD74/CXCR4 axis may represent a candidate mediator of senescence-associated tumor–immune interactions and a potential therapeutic target.

## 1. Introduction

Colorectal cancer (CRC) is one of the leading causes of cancer-related morbidity and mortality worldwide [1]. Its development is commonly characterized by a multistep progression from normal colonic mucosa through adenomatous precursor lesions to invasive carcinoma. This adenoma–carcinoma sequence is driven not only by the progressive acquisition of genetic and epigenetic alterations in epithelial cells but also by the dynamic remodeling of the surrounding immune and stromal microenvironment [2]. However, whether the accumulation of cellular senescence plays a role in tumor initiation, progression, and therapeutic response remains unclear.

Cellular senescence is a stress-responsive state characterized by durable cell-cycle arrest, resistance to apoptosis, metabolic and chromatin remodeling, and altered intercellular communication [3]. Senescence can be induced by telomere attrition, oncogene activation, DNA damage, oxidative stress, mitochondrial dysfunction, and exposure to anticancer therapy [4]. It was initially recognized as an intrinsic tumor-suppressive mechanism because it limits the proliferation of damaged or oncogenically stressed cells. In premalignant lesions, oncogene-induced senescence may restrict the expansion of transformed epithelial cells and delay malignant progression. However, persistent senescent cells can exert context-dependent tumor-promoting effects, particularly through the senescence-associated secretory phenotype (SASP) [5,6,7].

The SASP comprises a heterogeneous repertoire of cytokines, chemokines, growth factors, extracellular matrix-remodeling enzymes, and other secreted mediators [8,9]. Frequently reported SASP components include IL-6, CXCL8, CXCL1, CXCL2, CCL2, matrix metalloproteinases, and insulin-like growth factor-binding proteins [10]. Through autocrine and paracrine signaling, SASP factors can reinforce senescence, recruit or reprogram immune cells, remodel the extracellular matrix, stimulate epithelial plasticity, and promote tumor-cell invasion [9]. The composition and biological consequences of the SASP vary according to the cell type, senescence inducer, tissue context, and disease stage, which may partly explain the paradoxical tumor-suppressive and tumor-promoting roles attributed to senescence [11].

The interaction between senescence-associated epithelial states and immune-cell dysfunction may create a self-reinforcing tumor-promoting circuit. SASP factors released by epithelial or stromal cells can recruit and reprogram myeloid and lymphoid populations, whereas senescent or dysfunctional immune cells may fail to efficiently eliminate premalignant and malignant cells [12]. In addition, senescence-associated signaling may alter the balance between immune surveillance and immune suppression within specific tumor niches [13]. Nevertheless, the cellular sources, communication pathways, and spatial organization of these senescence-associated programs across the normal–adenoma–carcinoma continuum remain poorly defined.

In this study, we integrated single-cell RNA-sequencing and spatial transcriptomic datasets spanning normal colorectal mucosa, adenoma, and primary CRC. We aimed to: (1) determine whether senescence-associated transcriptional activity increases along the normal–adenoma–carcinoma sequence; (2) identify the epithelial, immune, and stromal cell populations in which these programs are most prominent; (3) characterize stage-specific receptor–ligand interactions involving senescence-high cells; and (4) define the spatial niches occupied by senescence-associated cell states within CRC tissues. By combining multiple senescence signatures, SASP scoring, cell–cell communication inference, and spatial neighborhood analysis, we sought to construct a cell-type-resolved and spatially informed model of senescence during colorectal tumor evolution.

## 2. Results

### 2.1. Overview of the scRNA-Seq Dataset and Senescence Signature Landscape

Single-cell RNA sequencing data were obtained from four patients spanning the adenoma-to-carcinoma progression sequence of CRC, comprising 39,365 cells with 12 tissue-derived samples covering four tissue categories: normal mucosa (*n* = 11,886 cells), paired adjacent para-cancerous tissue (para-cancer, *n* = 3290 cells), adenoma (including two additional independently sampled adenoma specimens from Patient3, merged as “adenoma”; total *n* = 11,712 cells), and carcinoma (*n* = 12,477 cells). Cells were annotated into 18 cell types using canonical marker-gene-based classification, including epithelial/tumor-lineage populations (cancer-associated epithelial, tumor stem cell-like cell, secretory epithelial, colonocyte, BEST4+ colonocyte, goblet, stem/TA cell), stromal cells (fibroblast), myeloid cells (monocyte, macrophage, mast), and lymphoid populations (CD4^+^ T cell, CD8^+^ T cell, Treg, NK cell, B cell, plasma, plasmacyte; Figure S1).

Of the 12 curated senescence/SASP/DNA damage response (DDR)-related gene signatures examined, 11 retained ≥ five detectable genes after filtering (coverage range 86.4–100%; e.g., SenMayo: 121/125 genes, 96.8%; CellAge core set: 270/278 genes, 97.1%; fibrotic SASP and T-cell senescence/exhaustion signatures: 100% coverage), confirming the broad applicability of established senescence gene panels to this dataset.

### 2.2. Gene-Set-Level Senescence and SASP Scores Vary Consistently Across the Disease Progression Sequence

**Figure 1:**
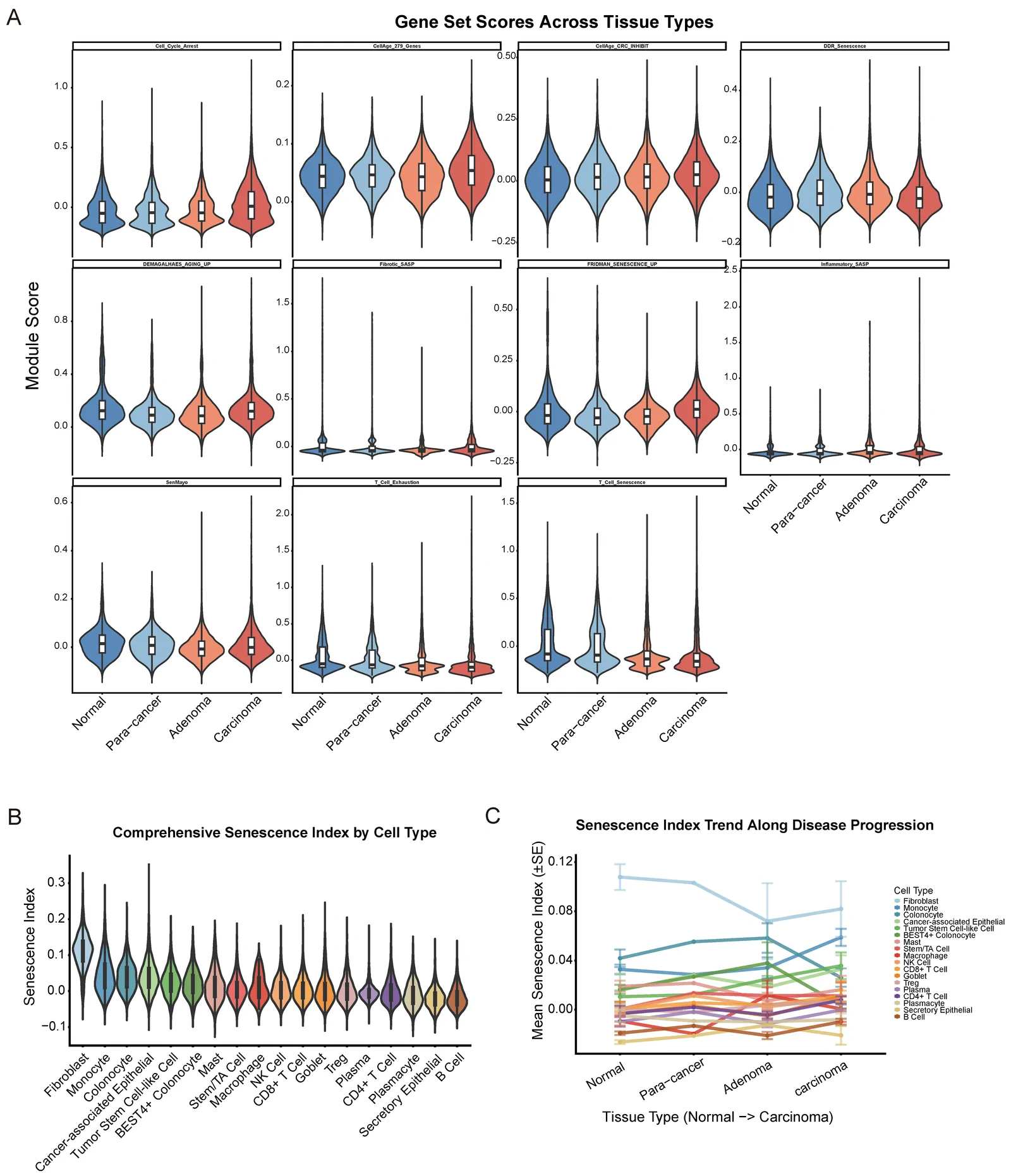
Senescence index landscape across cell types and tissue stages in CRC progression. (**A**) The module score of 11 curated senescence-associated gene sets across normal (blue), para-cancer (baby blue), adenoma (orange), and carcinoma (maroon) tissues. Gene sets include: SenMayo, T cell exhaustion, T cell senescence, DEMAGALHAES aging up, fibrotic SASP, fridman senescence up, inflammatory SASP, cell cycle arrest, CellAge 279 genes, CellAge CRC inhibit, and DDR senescence. Boxes show the interquartile range (IQR) with median; whiskers extend to 1.5× IQR. (**B**) Violin plot showing the distribution of the composite Senescence Index for each major cell type across all tissue samples combined. (**C**) Mean Senescence Index (±standard error) for each major cell type across the four tissue stages (normal, para-cancer, adenoma, and carcinoma).

Visualization of all 11 curated senescence/SASP/DDR module scores across the four tissue stages (Figure 1A) revealed tissue-stage-dependent distributions for each gene set, with distinct patterns observed among the SASP-associated signatures (SenMayo, fridman senescence-up, inflammatory SASP, fibrotic SASP), the cell-cycle-arrest/DDR signatures (cell cycle arrest, DDR senescence, CellAge core and CRC-inhibitory subsets), and the lymphocyte dysfunction signatures (T cell exhaustion, T cell senescence). Across nearly all gene sets, carcinoma tissue exhibited the broadest score distribution and the highest upper-quartile values relative to normal mucosa, while adenoma tissue showed intermediate or, for several SASP-related signatures, transiently elevated scores relative to normal and para-cancer tissues—consistent with a biphasic pattern in which senescence-associated transcriptional programs are already partially activated at the pre-malignant (adenoma) stage and further amplified upon progression to invasive carcinoma.

### 2.3. Fibroblasts and Myeloid Cells Exhibit the Highest Senescence Score

Using the top-decile threshold of the composite Senescence Index, the proportion of high-senescence-score cells varied markedly across cell types. Stromal fibroblasts exhibited by far the highest high-senescence-score cell fraction, followed by monocytes and colonic epithelial populations (colonocyte; cancer-associated epithelial; tumor stem cell-like cell; BEST4+ colonocyte; Figure 1B,C). In contrast, lymphoid populations displayed comparatively low senescence scores (CD8^+^ T cell; CD4^+^ T cell; NK cell; Treg), as did plasma cell lineages (plasma; plasmacyte) and B cells (Figure 1B). This pattern is consistent with the established biology of fibroblast senescence as a dominant contributor to the tissue SASP microenvironment and suggests that myeloid and epithelial/tumor-lineage cells—rather than lymphocytes—represent the principal senescence-associated cell reservoirs within the colorectal tumor microenvironment. 

**Figure 2:**
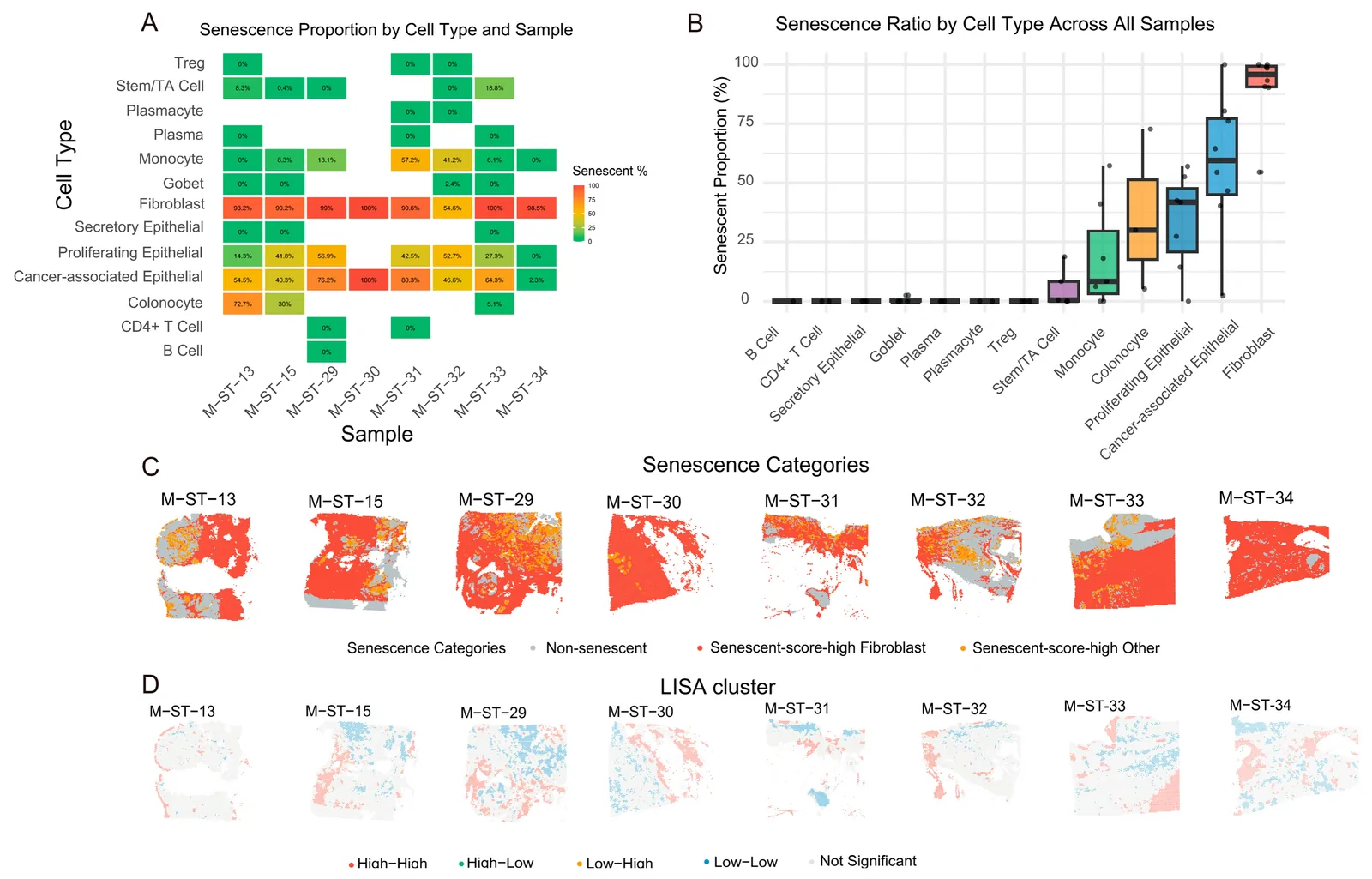
Spatial organization of senescence and LISA-defined clustering across eight CRC samples. (**A**) Heatmap showing the proportion of high-senescence-score cells within each cell type for each sample. Fibroblasts and epithelial lineages contribute substantially to the senescent compartment, although their relative contributions vary markedly across samples. (**B**) Box plot summarizing the senescence ratio by cell type across all samples. Fibroblasts exhibit the highest average senescence proportion, followed by epithelial populations (cancer-associated epithelial, tumor stem cell-like cell, secretory epithelial), while immune cell types such as B cells, CD4^+^ T cells, and Tregs show relatively lower senescence frequencies. (**C**) Spatial distribution of senescence categories (Non-senescent, high-senescence-score fibroblast, high-senescence-score other) across the eight CRC samples. High-senescence-score cells are not randomly distributed; instead, they show a tendency to localize toward the tumor periphery and stromal compartments. (**D**) LISA cluster maps for the senescence index across the eight CRC samples. High–High clusters (red) are predominantly enriched at the tumor periphery, invasive front, and adjacent stromal regions, while the tumor core is largely dominated by Low–High, High–Low, Low–Low or Not Significant clusters.

An abundance of high-senescence-score fibroblasts was observed in spatial transcriptomic cancerous samples (range: 54.6–100%, Figure 2A–C). Across all eight primary CRC spatial samples, local indicators of spatial association (LISA)-defined High–High clusters for the senescence index appeared to localize preferentially to the tumor periphery, invasive front, and adjacent stromal/immune-infiltrated regions (87.3%, range: 79.1–94.2%), whereas the tumor core contained only sparse (12.7%, range: 5.8–20.9%) or no significant High–High clusters (Fisher’s exact test, *p* < 0.05 per sample; Figure 2D). This spatial pattern suggests that senescence-associated transcriptional programs may be non-uniformly distributed across the tumor microenvironment, with a tendency toward enrichment at the tumor–host interface. However, we note that this descriptive pattern alone does not establish functional compartmentalization, and quantitative validation in larger spatial cohorts will be necessary to confirm this trend. 

Several technical considerations warrant caution when interpreting these spatial observations. Because each 10× Visium spot contains approximately 40–50 cells and non-proliferative senescence-associated cells contribute proportionally fewer transcripts than actively dividing cells, senescence-associated transcripts may be diluted within spots dominated by proliferating cells. This technical effect, rather than negative selection against high-senescence-score cells, may partly explain the observed spatial pattern. Higher-resolution spatial platforms (e.g., Visium HD) will be required to distinguish whether the apparent underrepresentation of senescence-associated transcriptional programs in highly proliferative regions reflects genuine negative selection against high-senescence-score cells or is primarily a consequence of transcript dilution. Accordingly, the spatial enrichment at the tumor periphery should be viewed as a hypothesis-generating observation rather than a definitive demonstration of senescence-driven spatial niche formation.

### 2.4. Global Senescence Scores Increase Progressively Along the Normal–Adenoma–Carcinoma Sequence

**Figure 3:**
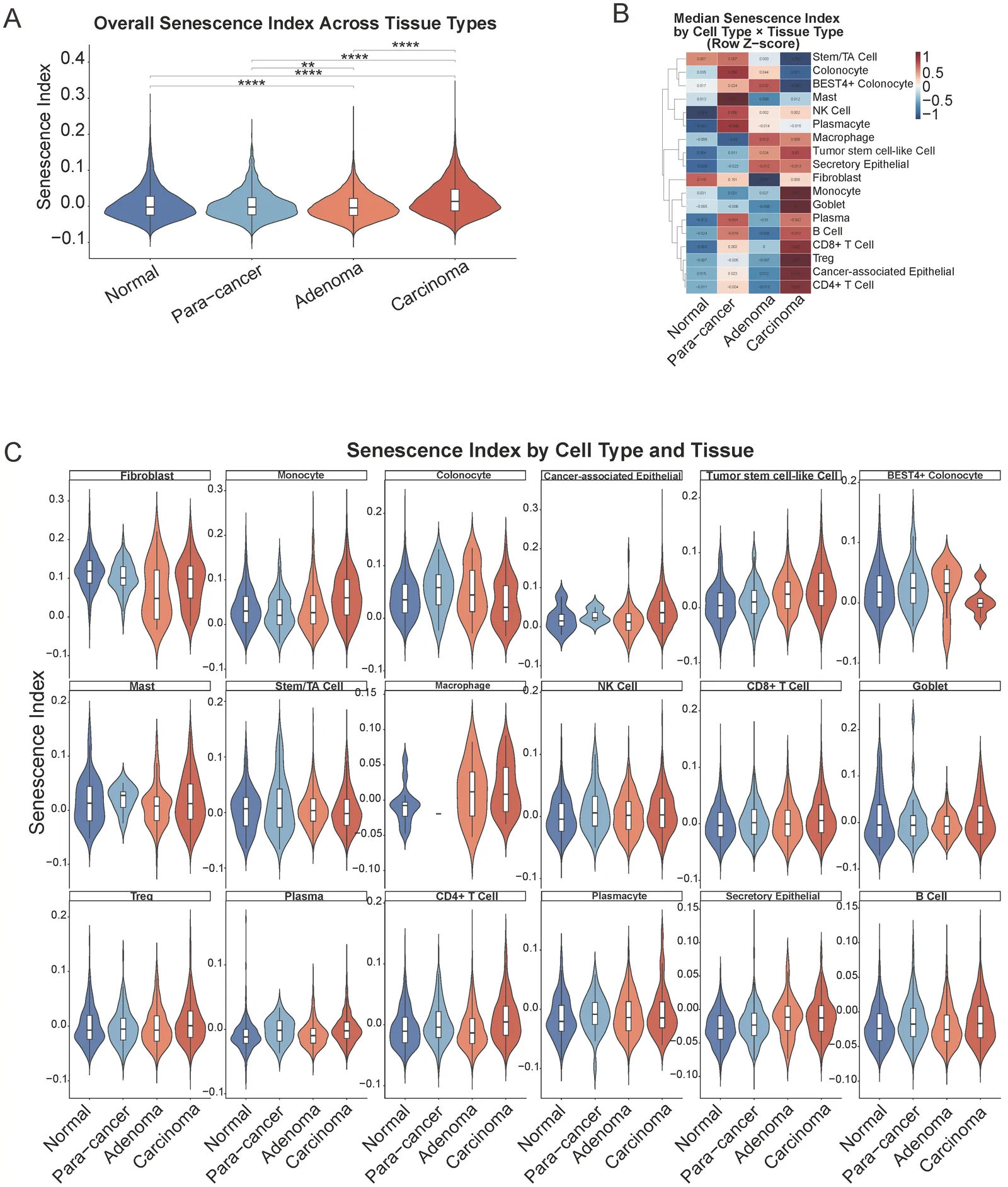
Cell-type-specific senescence dynamics and transcriptional programs along colorectal cancer progression. (**A**) Overall Senescence Index across the four tissue types (normal, para-cancer, adenoma, and carcinoma) based on single cells. Carcinoma tissue displayed the highest median Senescence Index, consistent with a progressive increase in senescence score along the adenoma–carcinoma sequence. (**B**) Heatmap of the median Senescence Index across cell types and tissue stages. The color scale represents row-scaled Z-scores, with red indicating higher senescence scores relative to the row mean and blue indicating lower senescence scores. (**C**) Violin plot showing Senescence Index stratified by cell type and tissue origin. Each sub-panel represents one cell type, with the Senescence Index displayed for normal, para-cancer, adenoma, and carcinoma tissues. ** *p* < 0.01, **** *p* < 0.0001.

Comparison of the composite Senescence Index across the four tissue stages (normal, para-cancer, adenoma, and carcinoma), performed on the globally scored dataset to preserve cross-tissue comparability, revealed a progressive increase in the senescence score from normal mucosa through adenoma to carcinoma (Figure 3A). This trend was particularly evident within the epithelial compartment, suggesting that tumor-intrinsic senescence escape accompanies malignant progression.

### 2.5. Epithelial Populations Show the Most Disease-Stage-Dependent Increase

To further dissect the cellular weights that were associated with the escalating trend from normal to carcinoma, we deconvoluted the senescent trend of each cell type. Notably, when this same per-cell-type Senescence Index was decomposed across the four tissue stages, the malignant epithelial compartments (cancer-associated epithelial, tumor stem cell-like cell) and colonocyte populations showed the most pronounced tissue-stage-dependent increase in the Senescence Index toward carcinoma, whereas fibroblasts, despite their high absolute per-cell senescence score, showed comparatively stable index values across tissue stages (Figure 3B,C). In addition, the trend of the Senescence Index at the unpaired sample level is generally consistent with that of the single cells (Figure S2A). Further paired-sample-level comparisons revealed statistically significant differences in Senescence Index across normal tissue, adenoma, and carcinoma for tumor stem cell-like cells and CD8^+^ T cells (Friedman test, χ^2^ = 6.00, df = 2, *p* = 0.0498; Figure S2B,C). However, it is important to emphasize that the paired-sample-level comparison included only three patient samples (patient1, patient2, and patient3; Table S1), representing a very limited sample size. With such a small sample size, the Friedman test has low statistical power, and the significance level (*p* = 0.0498) is close to the 0.05 threshold and may not be robust. Furthermore, the small sample size limits our ability to adequately assess inter-patient heterogeneity and to rule out the potential influence of individual variability on the observed results. Therefore, although these findings suggest that tumor stem cell-like cell senescence levels in all three patient samples may change significantly along the normal–adenoma–carcinoma sequence, they should be regarded as exploratory preliminary observations that require validation in larger patient cohorts to obtain more reliable and generalizable conclusions. In short, this cell-type-resolved analysis reinforces our revised model: the rise in the whole-tissue senescence score during CRC progression may be driven primarily by the emergence and expansion of intrinsically senescent malignant epithelial cells, not by an increase in fibroblast senescence.

### 2.6. CellChat Analysis Identifies High-Senescence-Score Malignant Epithelial Cells, Not Fibroblasts, as the Dominant Source of Senescence-Associated Intercellular Signaling, Converging on the Macrophage Migration Inhibitory Factor (MIF) and Midkine (MK) Pathways

**Figure 4:**
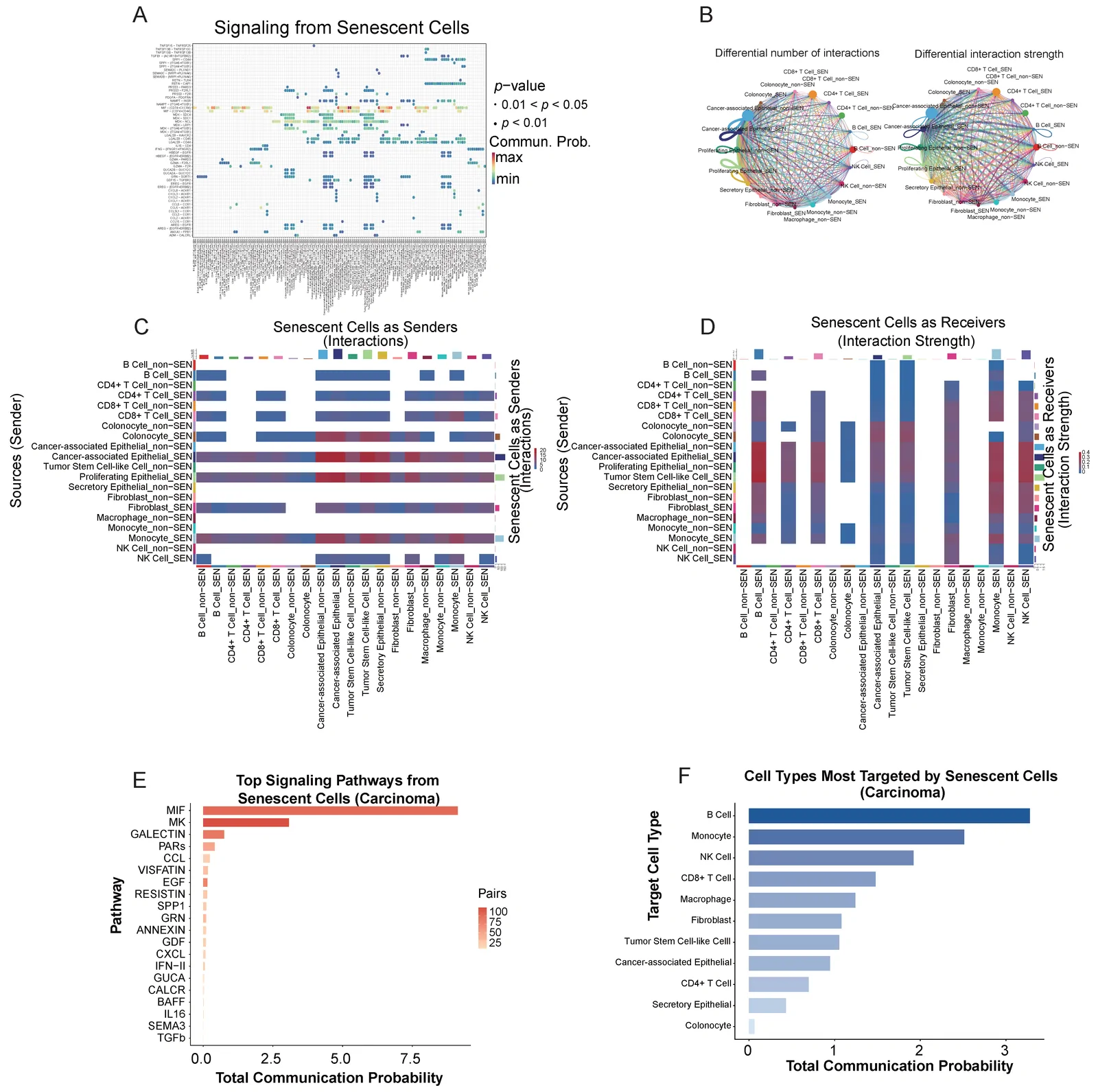
Senescence-associated intercellular communication network in the colorectal carcinoma microenvironment inferred by CellChat. (**A**) Bubble plot of significant ligand–receptor interactions originating from senescent (SEN) cell subpopulations in carcinoma tissue. Each row represents a ligand–receptor pair, and columns represent sender–receiver cell pairs (only the top interactions are shown). Dot color indicates communication probability (darker = higher), and dot size corresponds to statistical significance. (**B**) CellChat-inferred cell–cell communication networks showing the number of inferred ligand–receptor interactions from each senescent sender subpopulation to each target cell subset. (**C**) Heatmap displaying the total interaction strength (summed communication probability) for the same sender–target pairs as in (**B**). Malignant epithelial populations (cancer-associated epithelial_SEN, tumor stem cell-like cell_SEN) rank among the strongest outgoing signal sources. (**D**) Heatmap showing the number of inferred interactions in which each cell subset acts as a receiver of senescence-associated signals from the indicated sender subpopulations. (**E**) Bar plot of the top signaling pathways emanating from high-senescence-score cells, ranked by total communication probability. MIF and MK are the two overwhelmingly dominant pathways, followed by GALECTIN, PARs, and CCL signaling. (**F**) Bar chart summarizing the total communication probability received by each major cell type from all senescent senders in carcinoma. B cells, monocytes, and NK cells are the top-ranked targets.

High-senescence-score malignant epithelial cells in carcinoma tissues may not only dominate numerically but may also play a key role in microenvironmental communication. To further validate this inference at the level of intercellular signaling, we used CellChat to analyze the communication patterns of high-senescence-score cell subpopulations in the tumor microenvironment. Pathway-level aggregation of CellChat communication probabilities specific to senescent-cell subpopulations identified MIF (MIF–(CD74+CD44) and MIF–(CD74+CXCR4); total probability = 9.13, 104 significant ligand–receptor pairs) and MK (MDK–(ITGA4+ITGB1), MDK–(ITGA6+ITGB1), MDK–LRP1, MDK–NCL, MDK–SDC1/SDC4; total probability = 3.07, 124 pairs) as the two dominant signaling pathways associated with senescent-cell-derived communication in the carcinoma microenvironment, followed at a substantially lower magnitude by GALECTIN (0.75), PARs (0.41), CCL (0.24), and EGF (0.15) (Figure 4A–E). However, these results should be interpreted as exploratory and require direct experimental validation, including receptor inhibition or ligand neutralization studies. We therefore interpret these findings as generating a testable hypothesis rather than providing confirmatory evidence of a functional signaling axis.

Analysis of communication in source–target pairs revealed that high-senescence-score malignant epithelial populations (cancer-associated epithelial_SEN and tumor stem cell-like cell_SEN) constituted the dominant inferred sources of senescence-associated outgoing signaling when aggregated across the entire cell population,, targeting predominantly B cells (tumor stem cell-like cell _SEN → B cell_non-SEN, prob = 0.396; tumor stem cell-like cell_SEN → B cell_SEN, prob = 0.363; cancer-associated epithelial_SEN → B cell_non-SEN, prob = 0.362) and myeloid populations (tumor stem cell-like cell_SEN → macrophage_non-SEN, prob = 0.310; cancer-associated epithelial_SEN → macrophage_non-SEN, prob = 0.290), as well as NK cells and monocytes (Figure 4C,D). At the aggregate target level, B cells received the highest cumulative senescence-associated signaling input (47 significant pathways, total probability = 3.28), followed by monocytes (84 pathways, total probability = 2.51) and NK cells (59 pathways, total probability = 1.92), while CD8^+^ T cells, macrophages, and fibroblasts received comparatively lower but still substantial input (Figure 4E). 

We emphasize, however, that the CellChat-derived total communication probability scales with the number of cells in each source population, and our analysis did not include explicit normalization for cell-type abundance in these senescence-specific subset comparisons. The observed dominance of high-senescence-score epithelial populations is therefore attributable, at least in large part, to their marked numerical expansion in carcinoma tissue (24.7% of carcinoma cells) relative to high-senescence-score fibroblasts (0.42% of carcinoma cells). Consistent with this interpretation, high-senescence-score fibroblasts exhibited the highest per-cell senescence score of any lineage, yet their aggregate contribution to senescence-associated outgoing signaling was numerically modest. We therefore interpret these results as reflecting the population-level abundance-weighted signaling landscape of the tumor, rather than per-cell signaling potency.

Taken together, these findings suggest a model in which the paracrine senescence-associated communication network within colorectal carcinoma is shaped predominantly by the expanded abundance-weighted population of high-senescence-score tumor epithelial cells, with MIF and MK signaling representing the principal inferred mediators of epithelial-to-immune communication. We note, however, that (i) these inferences reflect the signaling potential based on the transcriptomic co-expression of ligand–receptor pairs rather than measured signaling activity; (ii) the analysis does not account for spatial proximity, post-translational regulation, or ligand–receptor stoichiometry; and (iii) definitive resolution of the relative contributions of epithelial versus fibroblast populations to senescence-associated paracrine signaling will require spatially resolved functional studies with equalized cell inputs.

## 3. Discussion

In this study, we characterized senescence-associated transcriptional states across the normal mucosa–adenoma–carcinoma sequence in colorectal tissues. Our findings indicate that senescence-associated programs are heterogeneous across cell types and spatial regions, rather than representing a uniform feature of colorectal tumorigenesis. Although a modest increase in senescence-related scores was observed during the transition from normal mucosa to adenoma, a more substantial increase was detected in carcinoma. This pattern was consistently supported by independent gene signatures (Figure 1A), suggesting that the observed trend is not dependent on a single gene set. Importantly, these results should be interpreted as evidence of increased senescence-associated transcriptional activity rather than definitive proof of irreversible cellular senescence, because transcriptomic signatures alone cannot fully distinguish senescence from stress responses, differentiation states, or cell-cycle alterations.

The gradual increase in senescence-associated activity across disease stages is biologically plausible in the context of CRC evolution. During early adenoma formation, oncogene activation and replication stress may induce a senescence-like response that acts as a barrier to malignant transformation [14]. This phenomenon is consistent with the concept of oncogene-induced senescence, whereby cells exposed to aberrant proliferative signaling undergo cell-cycle arrest to prevent the expansion of potentially transformed clones. However, as adenomas progress to carcinoma, persistent DNA damage, chromosomal instability, metabolic stress, hypoxia, and inflammatory signaling may promote the accumulation of senescence-associated states [15]. Thus, senescence may exert stage-dependent and context-dependent effects: it may initially constrain abnormal epithelial proliferation, while persistent senescence-associated cells and their secretory programs may later contribute to tumor-promoting inflammation, stromal remodeling, and immune dysfunction.

Malignant epithelial populations displayed elevated senescence-associated scores together with increased SASP activity, indicating that tumor cells may contribute directly to the inflammatory and remodeling features of the tumor microenvironment. Senescent cells are not necessarily metabolically inert; instead, they may remain highly secretory and actively influence neighboring cells through cytokines, chemokines, growth factors, and extracellular matrix-remodeling enzymes [16]. In this context, increased SASP activity may promote local inflammation, angiogenesis, epithelial plasticity, and tumor–stromal interactions [17].

A prominent finding of this study was the association between epithelial senescence-associated programs and changes in the immune microenvironment. Notably, and contrary to our initial hypothesis, the fibroblast fraction did not increase during malignant progression. T cell populations, particularly CD8^+^ and CD4^+^ T cell subsets, exhibited increased senescence-associated transcriptional scores during progression from normal tissue to carcinoma. These observations should be distinguished from canonical T-cell exhaustion. Increased expression of inhibitory receptors such as PDCD1, HAVCR2/TIM-3, LAG3, TIGIT, and CTLA4 is generally more indicative of chronic antigen-driven exhaustion than of senescence *per se* [18]. In contrast, immune-cell senescence is more closely linked to impaired proliferative capacity, cell-cycle arrest, replicative stress, and markers such as KLRG1 and B3GAT1/CD57 [19,20]. Therefore, the T cell populations identified in this study may represent overlapping senescence-associated and exhaustion-associated states, rather than a single homogeneous dysfunctional population. This distinction is clinically relevant because exhausted T cells may retain partial reversibility and responsiveness to immune-checkpoint blockade, whereas senescent T cells may require alternative strategies aimed at restoring proliferative competence or modifying the suppressive microenvironment.

Our cell–cell communication analysis further identified enhanced MIF signaling involving senescence-high cell populations and B-cell subsets expressing the CD74/CXCR4 receptor complex. MIF is a pleiotropic cytokine with established roles in inflammation, tumor progression, immune-cell recruitment, and macrophage polarization [21]. Its receptors, CD74 and CXCR4, are expressed on epithelial and immune cells, making this axis a plausible amplifier of senescence-associated inflammation during colorectal carcinogenesis. The observed enrichment of the MIF–CD74/CXCR4 axis raises the possibility that senescence-associated cells may influence B-cell behavior or other CD74^+^CXCR4^+^ immune populations through paracrine signaling. However, this pathway should be regarded as a candidate mechanism that requires validation using spatial colocalization, multiplex immunofluorescence, ex vivo co-culture systems, or perturbation experiments targeting MIF (e.g., imalumab), CD74, or CXCR4 (e.g., plerixafor, motixafortide).

Our findings have several translational implications. The dual role of senescence suggests that therapeutic strategies should be tailored to the disease stage and cellular context. In premalignant lesions, the premature elimination of senescent cells could theoretically disrupt a protective tumor-suppressive barrier. In established CRC, however, persistent senescence-associated cells and SASP signaling may contribute to chronic inflammation, stromal activation, immune dysfunction, and therapeutic resistance. Therefore, approaches targeting senescence in CRC may require selective rather than indiscriminate intervention. Potential strategies include senolytic therapies designed to eliminate harmful senescence-associated cells, senomorphic therapies aimed at suppressing deleterious SASP signaling, and combination approaches that modulate tumor-cell senescence-associated programs and immune dysfunction [22]. The MIF–CD74/CXCR4 axis may be particularly relevant in this context, although its therapeutic value remains to be established experimentally.

Several limitations should be acknowledged. First, transcriptomic senescence signatures do not provide definitive evidence of irreversible cell-cycle arrest. Orthogonal validation on formalin-fixed paraffin-embedded (FFPE) or fresh-frozen tissue sections from normal mucosa, adenoma, and carcinoma specimens using protein-level markers— such as p16, p21, and Ki-67 and, where feasible, SA-β-gal activity and co-staining with cell-type-specific markers (e.g., *EPCAM* for epithelial cells, *CD45* for immune cells)—to validate the cell-type-specific senescence patterns predicted by our analysis would strengthen the biological interpretation of the identified senescence-high populations. The small number of patients and the heterogeneity of tumor stage limit the generalizability of our findings. Larger, clinically well-annotated cohorts with matched normal, adenoma, and carcinoma samples will be required to determine whether the observed senescence-associated transcriptional programs are consistent across patients with different levels of baseline inflammation and tumor immune contexture. Second, spatial transcriptomic spots contain multiple cells, limiting the precise assignment of senescence-associated programs to individual cell types. Using multiplex immunohistochemistry (mIHC) or high-resolution spatial transcriptomics (e.g., Visium HD) to confirm the preferential enrichment of senescence signatures within tumor-associated spatial niches may eliminate interference from mixed cells. Third, ligand–receptor analyses are inferential and cannot establish direct signaling or causality. Performing in vitro co-culture experiments using CRC cell lines, senescent fibroblasts, and immune cells to test whether MIF secreted by senescent cells activates CD74/CXCR4 signaling and using CRISPR/Cas9 knockout or pharmacological inhibitors (e.g., anti-MIF antibodies, CD74-blocking peptides, or CXCR4 antagonists such as plerixafor) to test whether disruption of this axis reduces senescence-associated inflammation and tumor progression would validate the role of the MIF–CD74/CXCR4 axis in the senescence of CRC cells. Longitudinal sampling, organoid and patient-derived xenograft (PDX) models, genetically engineered mouse models, and functional perturbation experiments will be necessary to determine whether senescence-associated programs actively drive colorectal tumor progression or primarily reflect tumor evolution.

In conclusion, this study provides an integrated single-cell and spatial view of senescence-associated programs during colorectal tumorigenesis. Our results support a model in which senescence-associated transcriptional activity increases from normal mucosa to adenoma and carcinoma, with contributions from epithelial and immune compartments and preferential localization within tumor-associated spatial niches. Defining the functional roles of these programs, their spatial interactions, and their relationship to immune dysfunction may facilitate the development of more precise biomarkers and context-specific therapeutic strategies for CRC.

## 4. Materials and Methods

### 4.1. Single-Cell RNA Sequencing Data Acquisition and Preprocessing

The single-cell RNA-seq dataset GSE161277 [23] was downloaded from the Gene Expression Omnibus (GEO). A total of twelve tissue samples were collected from four untreated CRC patients with microsatellite stability. Additionally, samples of normal, para-cancer, adenoma, and cancer tissues were collected from the same patient who underwent radical surgery. The original study [23] was approved by the local ethics committee of West China Hospital, Sichuan University, with written informed consent collected. For each sample, the count matrix was loaded into R (version 4.4.1) using the *Read10×* function of the *Seurat* package (version 5) [24] and a Seurat object was created with the parameters min.cells = 3 and min.features = 200. Samples were then merged into a single object, and tissue-type annotations (normal, para-cancer, adenoma, and carcinoma) were assigned based on the sample identifiers.

### 4.2. Quality Control and Normalization

Cells were filtered by the number of detected genes (200 < *n* < 8000), total UMI (unique molecular identifier) counts (*n* < 40,000) and the percentage of mitochondrial reads (percent.mt < 25%). Data were normalized using the default log-normalization method with a scale factor of 10,000. Highly variable genes (*n* = 2000) were selected using the variance-stabilizing transformation (VST) method, and expression values were scaled while regressing out the effects of the total UMI count and mitochondrial gene percentage. Principal component analysis (PCA) was performed on the variable genes, with 30 dimensions computed.

### 4.3. Batch Effect Correction and Clustering

To mitigate inter-sample technical variation, Harmony (with group.by.vars = “sample”, theta = 2, 20 iterations) was applied to the PCA embeddings. The resulting Harmony reduction was used to construct a shared nearest-neighbor graph (20 dimensions) and clustering was performed at a resolution of 0.5. The data were then visualized using uniform manifold approximation and projection (UMAP) with the Harmony-corrected coordinates.

### 4.4. Cell Type Annotation

Cluster identities were inferred by examining the expression of well-established lineage markers: *EPCAM*, *KRT8*, and *KRT18* (epithelial cells); *COL1A1*, *DCN*, and *ACTA2* (fibroblasts); *CD68*, *CD163*, and *CSF1R* (monocytes/macrophages); *CD3D*, *CD8A*, and *CD4* (T cells); *NKG7*, *GNLY*, and *KLRB1* (NK cells); *CD19*, *MS4A1*, and *CD79A* (B cells); *JCHAIN* and *MZB1* (plasma cells); *CLEC10A* and *FCER1A* (dendritic cells); and *TPSAB1* and *CPA3* (mast cells). Manual annotation was performed by assigning each cluster to the cell type whose marker profile was most consistent. To avoid contamination by smooth-muscle cells, the fibroblast compartment was validated using *DCN*, *COL1A1*, and *PDGFRA*, and cells co-expressing high levels of *ACTA2* without the canonical fibroblast markers were excluded or assigned to a separate pericyte/smooth-muscle group.

### 4.5. Senescence Scoring 

To characterize cellular senescence programs at single-cell resolution, 12 curated gene signatures relevant to senescence, SASP, DDR, and T/NK cell dysfunction were compiled from established databases and publications, including SenMayo [25], DEMAGALHAES aging-up, Fridman senescence-up, CellAge (279-gene core set and CRC-specific inhibitory subset) [26], inflammatory and fibrotic SASP subsets [9], T-cell senescence/exhaustion signatures [19,20], NK-cell senescence markers, cell-cycle arrest genes, and a DDR-associated senescence signature [27]. The gene sets were filtered to retain only genes detected in the expression matrix, and signatures retaining fewer than five genes after filtering (NK_cell_senescence) were excluded from downstream scoring, yielding 11 final gene sets (range: 10–270 genes per set; overall gene coverage 86–100% of the original curated lists).

Module scores were calculated for each retained gene signature across all cells (pooled across tissue types and patients) using the *AddModuleScore* function in *Seurat* (version 5), with control gene sets drawn from 24 expression bins (nbin = 24) matched for average expression to minimize confounding by the transcript detection depth (seed = 42 for reproducibility). Computing module scores on the full pooled dataset, rather than within tissue-stratified subsets, ensured that the expression-matched control gene pools were derived from a common background, thereby preserving the cross-tissue comparability of the resulting scores. For patient-level aggregated analysis, per-cell senescence scores were averaged within each patient/sample, and comparisons were performed using the Wilcoxon rank-sum test with patients as biological replicates.

A composite Senescence Index was derived for each cell as the arithmetic mean of five core senescence-associated module scores (SenMayo, fridman senescence-up, CellAge core 279-gene set, cell cycle arrest, and DNA damage response signatures), selected to jointly capture the SASP secretory activity, cell-cycle exit, and genotoxic stress response components of the senescence phenotype.

### 4.6. Identification of High-Senescence-Score Cell Populations

To determine the optimal threshold for classifying senescent cells, we performed a sensitivity analysis across a range of percentile thresholds (0.70 to 0.95, in increments of 0.05) applied to the Senescence Index. For each threshold, cells with senescence scores above the corresponding percentile cutoff were classified as senescent. We evaluated each threshold based on (1) the overall chi-square test significance across tissue types (normal, para-cancer, adenoma, and carcinoma), (2) the number of cell types showing significant differences in senescent cell proportions across tissues, and (3) the biological plausibility of the identified senescent cell populations. After comprehensive evaluation, the 0.90 percentile was selected as the primary threshold, as it provided the optimal balance between statistical power and biological specificity. The proportion of high-senescence-score cells was calculated within each of the 18 annotated cell types to identify populations enriched for the senescence phenotype.

### 4.7. Tissue-Stage Stratified Comparison of Senescence Scores

To evaluate the senescence dynamics along the adenoma-to-carcinoma progression sequence, cells were stratified into four tissue categories—normal, para-cancer (adjacent non-tumor), adenoma, and carcinoma—using the merged tissue annotation (tissue_merged). For each cell type and for the overall cell population, the composite Senescence Index and individual module scores were compared across tissue categories using pairwise two-sided Wilcoxon rank-sum tests, given the non-normal distribution of module scores. The cell-type-by-tissue composition was additionally cross-tabulated to assess whether shifts in the senescence score across tissue stages were attributable to changes in the underlying cellular composition (e.g., stromal or myeloid cell infiltration) or intrinsic per-cell-type increases in senescence signaling.

### 4.8. Cell–Cell Communication Analysis (CellChat)

To identify candidate intercellular signaling pathways associated with high-senescence-score cells in the carcinoma microenvironment, we performed ligand–receptor interaction analysis using CellChat (version 1.6.1) [28]. Within carcinoma-derived cells, each annotated cell type (restricted to CD4^+^ T cell, CD8^+^ T cell, monocyte, NK cell, B cell, cancer-associated epithelial/tumor stem cell-like cell/secretory epithelial, colonocyte, fibroblast, and macrophage; populations with insufficient cell numbers were excluded) was further stratified into “Senescent” and “Non-Senescent” subgroups based on the top-decile Senescence Index threshold defined above, generating composite communication labels (e.g., “fibroblast_senescent”, “fibroblast_nonsenescent”). A CellChat object was constructed using these composite labels, and the cell–cell communication probabilities were inferred using the standard CellChat workflow (over-expressed gene/interaction identification, communication probability computation, and pathway-level aggregation), using the human CellChatDB ligand–receptor reference database. Outgoing and incoming signaling strength specific to senescent subpopulations were extracted and ranked to identify the top target cell types and signaling pathways associated with senescent-cell-derived communication.

### 4.9. Spatial Transcriptomics Analysis

Eight primary CRC (10× Genomics Visium Spatial Gene Expression platform) were retrieved from GSE294385 [29]. Downstream analysis was performed in R using *Seurat* (version 5). Single-cell senescence annotations were projected onto the spatial map using spacexr’s (version 2.2.1) function *RCTD*. Briefly, a signature matrix was constructed by computing the average expression of each cluster’s top differentially expressed genes (by fold change). Deconvolution was performed with default parameters to estimate the abundance of each cell type at each Visium spot.

For each of the eight primary CRC Visium samples, four spatial compartments were delineated using a combined histopathological and transcriptomic approach: (1) tumor periphery, defined as the outermost 500 μm band of the carcinoma cell mass adjacent to the tumor–stroma interface; (2) invasive front, defined as the interface zone where malignant epithelial cells directly abut or intermingle with non-neoplastic stromal elements; (3) adjacent stromal/immune-infiltrated region, defined as the non-neoplastic compartment within 500 μm outside the invasive front (enriched for fibroblasts, immune cells, and endothelial cells); and (4) tumor core, defined as the central carcinoma region >1000 μm inward from the invasive front, characterized by confluent malignant glands with minimal intervening stroma. Histopathological annotation was performed by board-certified pathologists on H&E images, and was cross-validated by marker gene expression: epithelial/tumor spots were required to express markers such as *EPCAM* and *KRT8* above the 75th percentile, while stromal/immune spots were required to express markers such as *COL1A1*, *PTPRC*, *CD4*, *CD8*, and *PECAM1* above the 75th percentile. Discordant spots were individually reassigned based on combined evidence.

To identify spatially coordinated patterns of cellular senescence, we performed LISA analysis on senescence scores derived from spatial transcriptomics data. The LISA framework classifies each spatial location (spot) into one of five categories based on the local spatial autocorrelation between its own senescence score and the mean senescence score of its spatial neighbors: High–High (local and neighbor scores above the global mean), Low–Low (both below), Low–High (local below, neighbors above), High–Low (local above, neighbors below), and Not Significant (no statistically significant spatial association). For quantitative comparison, the proportion of senescence-associated High–High clusters within the peripheral/invasive front/adjacent stromal/immune-infiltrated region was compared to that within the tumor core for each sample using Fisher’s exact test. Samples were analyzed individually, and results were summarized across samples. *p* < 0.05 was considered statistically significant.

Briefly, for each Visium spot, we constructed a spatial weights matrix using k-nearest neighbors (*k* = 8, based on the Euclidean distance between spot coordinates). The senescence score for each spot was derived from a curated senescence gene set using Seurat’s *AddModuleScore* function, yielding the column Senescence_Score1 in the metadata. A permutation-based approach (*n* = 999 permutations) was employed to assess the statistical significance of local spatial association, with false discovery rate (FDR) correction applied across all spots. Spots were subsequently assigned to LISA clusters as described above. The resulting LISA cluster annotation was added to the Seurat object metadata for downstream visualization.

### 4.10. Immune Senescence Profiling

Within the immune compartment, T cells and myeloid cells were further subclustered to identify senescent phenotypes. For T cells, markers of exhaustion (*PDCD1*, *CTLA4*, and *LAG3*) and senescence (*KLRG1*, *CD57*/*B3GAT1*) were examined. Myeloid cells were assessed for senescence-associated markers such as *CDKN2A* and inflammatory SASP genes. The proportion of senescent immune cells was calculated per sample and compared across tissue stages using a linear mixed-effects model, with patient identity as a random effect. Correlations between the epithelial senescence scores and immune senescence proportions were evaluated using Spearman’s rank correlation.

### 4.11. Statistical Analysis

Statistical analysis was performed using R version 4.4.1. All tests were two-sided, and a *p*-value < 0.05 was considered statistically significant. Because some patients contributed samples to multiple tissue types (e.g., normal, adenoma, and carcinoma from the same patient), we first assessed the data structure to determine whether comparisons involved paired or independent observations. For comparisons involving paired observations from the same patients, we used the paired Wilcoxon signed-rank test (two-group comparisons) or the Friedman test (three-group comparisons). For comparisons involving independent samples derived from different patients, the unpaired Wilcoxon rank-sum test was used. Given the limited sample size in sample-level comparisons, formal hypothesis testing was often underpowered. In such cases, we relied primarily on descriptive and trend-based interpretation rather than *p* values: we report the direction and magnitude of differences, the proportion of patients exhibiting a consistent directional change, and individual patient trajectories where applicable. A consistent directional trend observed across the majority of patients was interpreted as supportive evidence, with the explicit acknowledgment that these exploratory findings require validation in larger independent cohorts.

### 4.12. Data Visualization

All UMAP plots, violin plots and bar graphs were generated using Seurat’s *DimPlot*, *FeaturePlot*, and *VlnPlot* functions, as well as *ggplot2* package (version 4.0.3). Figures were exported as vector-graphics PDFs.

## Data Availability

The datasets generated during this study are fully available within the article and Supplementary Materials. The single-cell RNA-seq and spatial transcriptomic matrices are available in the GEO Repository (https://www.ncbi.nlm.nih.gov/geo/ accessed on 8 June 2026) under accession numbers GSE161277 and GSE294385, respectively.

## Supplementary Materials

The following supporting information can be downloaded at: https://www.mdpi.com/article/10.3390/ijms27219799/s1.
