# Supplementary material for: In Silico Single-Cell Transcriptomic Analysis Reveals Senescence-Associated Programs’ Progress Along the Normal–Adenoma–Carcinoma Sequence in Colorectal Cancer
Source: Int J Mol Sci. 2026 Oct 31;27(21):9799. doi: 10.3390/ijms27219799 (PMC13762944; doi:10.3390/ijms27219799)
Supplement: Supplementary file 1 [file ijms-27-09799-s001.zip › Figure S2.pdf]

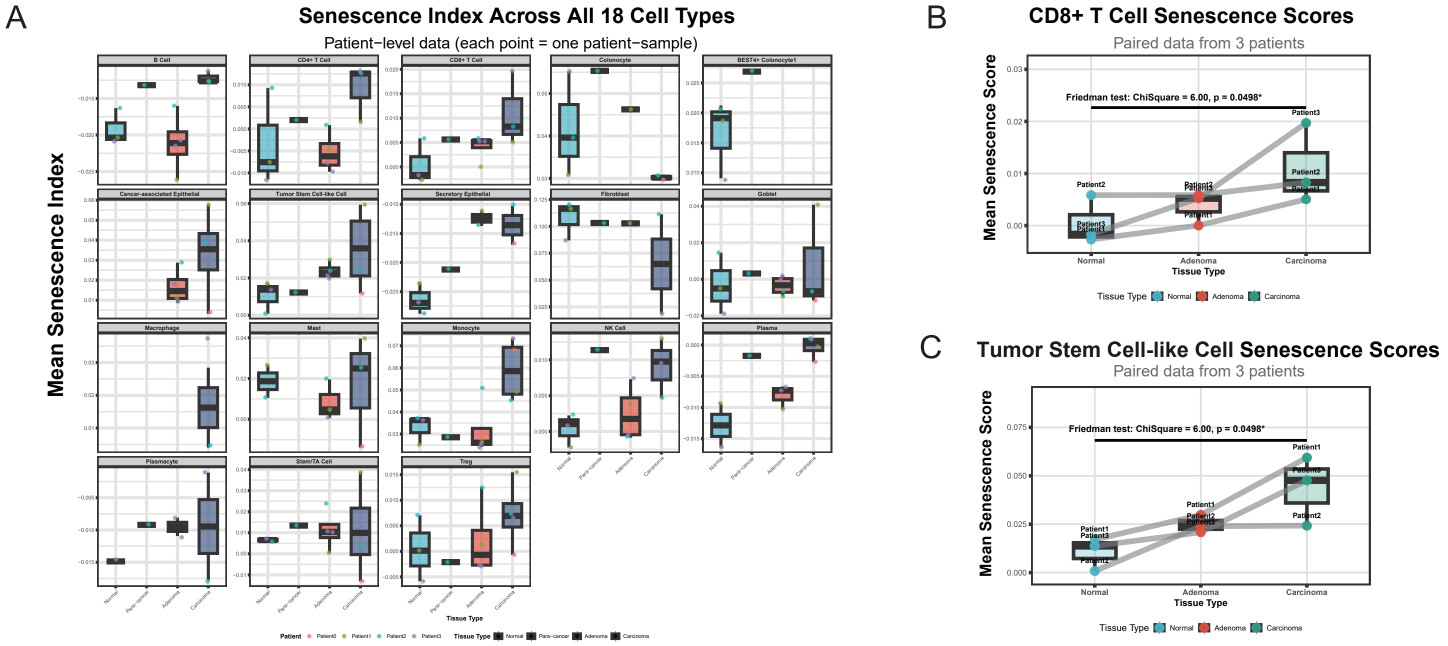


Figure S2. Senescence Index stratified by both cell type and tissue origin based on sample-level comparison. A. Each sub-panel represents one cell type, with the Senescence Index displayed for normal, para-cancer, adenoma, and carcinoma tissues. B. Friedman test analysis of CD8 T cell senescence scores across tissue types. Box plots showing mean senescence scores of CD8 T cells in Normal, Adenoma, and Carcinoma tissues from three paired patients ($n = 3$). Gray lines connect paired samples from the same patient. Colored points represent individual patient measurements. The Friedman test indicates a significant overall difference across tissue types ($\chi^2 = 6.00$, $p = 0.0498$). Box plots show median, interquartile range, and range. C. Friedman test analysis of tumor stem cell-like cell senescence scores across tissue types.
