# Supplementary material for: In Silico Single-Cell Transcriptomic Analysis Reveals Senescence-Associated Programs’ Progress Along the Normal–Adenoma–Carcinoma Sequence in Colorectal Cancer
Source: Int J Mol Sci. 2026 Oct 31;27(21):9799. doi: 10.3390/ijms27219799 (PMC13762944; doi:10.3390/ijms27219799)
Supplement: Supplementary file 1 [file ijms-27-09799-s001.zip › Figure S1.pdf]

A

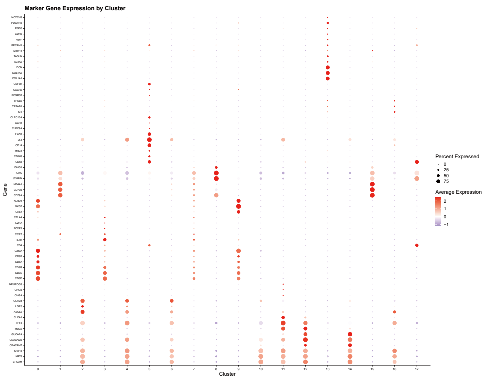

B

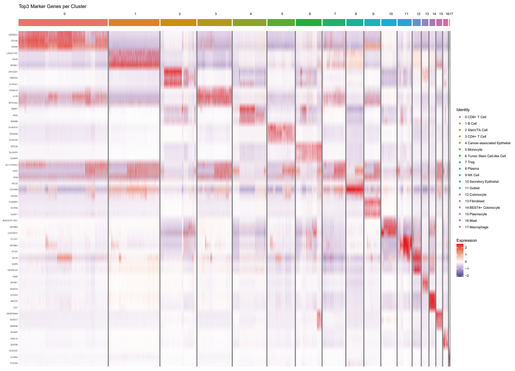

Figure S1. Clustering of the single cells. A. The expression of marker genes in each clusters. B. The top three marker genes for each cluster annotation.
